# 肝移植围手术期凝血功能管理：1例报告及文献复习

**DOI:** 10.3760/cma.j.cn121090-20241130-00508

**Published:** 2024-12

**Authors:** 瑶 唐, 嘉贤 吕, 常洁 蔡

**Affiliations:** 中山大学附属第一医院重症医学科，广州 510080 Department of Critical Care Medicine, The First Affiliated Hospital of Sun Yat-sen University, Guangzhou 510080, China

**Keywords:** 肝移植, 围手术期, 凝血, Liver transplantation, Perioperative period, Coagulation

## Abstract

**目的:**

探讨肝移植术前、术中、术后不同病理生理状态下凝血功能的变化特征及其相应的监测和临床管理策略，为其他医疗机构提供本中心的经验。

**方法:**

分析1例肝切除术后继发肝衰竭患者在肝移植围手术期的诊治过程，结合国内外相关指南共识，总结肝移植围术期的凝血功能特点以及管理策略。

**结果:**

本例报告中，肝移植围术期不同病程阶段病理生理机制差异明显，对应的凝血功能变化复杂难测。围术期各阶段的凝血特点可概括为术前血小板与凝血因子缺乏、术中低凝与纤溶亢进、术后凝血功能恢复不全以及整个过程中出血与血栓形成的风险并存，需根据凝血系统、血小板计数、血栓相关检验指标及反映出凝血系统整体功能如血栓弹力图的动态变化予目标导向性输注血制品、抗凝及抗纤溶等治疗，在动态监测相关指标前提下予以恰当、适度的治疗维持促凝、抗凝、纤溶三大系统间的相对稳态，既避免了因低凝导致的重要脏器出血，也降低了围术期出现血栓并发症的风险，精准的监测与个体化治疗可以改善患者预后。

**结论:**

肝移植围手术期的凝血功能管理复杂而多变，需要加强监测并予以积极有效的干预措施。本研究为终末期肝病患者围手术期的诊治提供了重要参考，并总结了有效的管理经验。

## Abstract (en)

**Objective:**

To investigate the characteristics of changes in coagulation function in different pathophysiological states during the preoperative, intraoperative, and postoperative phases of liver transplantation, along with its corresponding monitoring and clinical management strategies, and to provide our center's experience to other medical institutions.

**Methods:**

By reporting and analyzing the diagnosis and treatment process of a patient with secondary liver failure after hepatectomy in the perioperative period of liver transplantation, we summarize the characteristics of coagulation function in the perioperative period of liver transplantation as well as the management strategies, taking into account the consensus of relevant domestic and international guidelines.

**Results:**

In this case report, the pathophysiologic mechanisms of the different stages of perioperative liver transplantation differed significantly, and the corresponding changes in coagulation function were complex and difficult to measure. The coagulation characteristics of the perioperative stages can be summarized as preoperative platelet and coagulation factor deficiencies, intraoperative hypocoagulation and hyperfibrinolysis, postoperative incomplete recovery of coagulation function, and the coexistence of the risk of hemorrhage and thrombosis throughout the entire process, which should be treated with goal-oriented transfusion of blood products, anticoagulation, and antifibrinolysis, according to dynamic changes of the coagulation system, platelet counts, thrombus-related test indexes, and the overall function of the coagulation system such as the thromboelastogram. Under the premise of dynamic monitoring of relevant indicators, appropriate and moderate treatment should be given to maintain the relative homeostasis among the three systems of coagulation promotion, anticoagulation, and fibrinolysis, which not only avoids bleeding of vital organs caused by hypocoagulation, but also reduces the risk of perioperative thrombotic complications, and accurate monitoring and individualized treatment can improve the prognosis of the patients.

**Conclusion:**

Coagulation management in the perioperative period of liver transplantation is complex and variable, requiring enhanced monitoring and active and effective interventions. This study provides an important reference for the perioperative diagnosis and treatment of patients with end-stage liver disease and summarizes the effective management experience.

肝移植是各种终末期肝病最为有效的治疗手段，绝大多数肝移植受者术前合并肝功能衰竭。肝脏作为调节凝血平衡的核心器官，绝大多数凝血因子都在肝脏合成代谢，肝移植围手术期往往合并凝血功能的紊乱[1]。

在正常机体中，为维持血流的稳态，促凝、抗凝、纤溶三大系统形成相对稳定的动态平衡，而伴随着肝功能的持续衰竭，肝脏合成的促凝、抗凝因子及纤溶酶原的数量及功能都随之降低，三者又达到了脆弱的低水平平衡[2]。

肝移植患者术前因普遍存在血小板水平及功能低下、肝脏合成凝血因子能力下降、合并门脉高压等因素，出血风险高，但同时因抗凝因子生成减少，血栓形成风险也高，出血与高凝两者并存[3]；术中及手术各期均有稀释性和（或）消耗性凝血障碍的可能，包括低体温、低钙、高乳酸等进一步导致凝血异常的因素存在；术后早期移植肝功能不全时仍有凝血功能障碍持续存在的可能，但过度纠正凝血功能会导致肝移植术后血管并发症的发生[4]。三者之间并非均匀的此消彼长，很大程度上这种脆弱的稳态一旦被破坏都可能给肝移植受者带来极大的风险及不良预后，因此整体而言，肝移植围手术期的凝血功能管理复杂而多变，需要加强监测并予以积极有效的干预措施[5]。以下所述病例的诊治过程充分展示了肝移植围手术期凝血紊乱的复杂多变和及时有效干预的重要性。

## 病例资料

患者，女，41岁，2023年3月起无明显诱因出现右上腹痛，增强CT示肝S4/8肿块（45 mm×48 mm）（图1A），考虑肝癌可能。于2023年5月12日入住我院肝外科。患者既往慢性乙型肝炎病史十余年，未规律诊治。入院查体见右上腹轻压痛，余无特殊。相关检验检查：甲胎蛋白（AFP）630 µg/L，EBV-DNA 1.07×104 IU/ml，血常规、肝肾功能、凝血基本正常；超声造影及腹部磁共振成像（MRI）均考虑原发性肝癌（图1B、C），术前行吲哚菁绿（ICG）清除试验提示肝脏储备功能尚可。遂于5月25日在全麻下行“扩大左半肝切除术+胆囊切除术”，术中见左外叶及左尾状叶萎缩，术后第2天患者神志嗜睡伴肝功能异常，考虑肝切术后肝功能不全伴肝性脑病。于术后第3天转入ICU，转入时患者烦躁不安，可简单遵嘱，但定向力及计算力明显异常，皮肤巩膜中度黄染，腹部切口干洁无渗血，上腹部轻压痛。动脉血气示代谢性酸中毒，血乳酸4.5 mmol/L；其他检验指标：PLT 42×10^9^/L，凝血酶原时间（PT）20.4 s，活化部分凝血活酶时间（APTT）51 s，纤维蛋白原（Fib）1.21 g/L，丙氨酸转氨酶（ALT）1618 U/L，总胆红素（TBil）156 µmol/L，血氨90 µmol/L（参考值11～32 µmol/L），肌酐由术前41 µmol/L升至98 µmol/L，12 h尿量<400 ml，提示肝功能和凝血功能明显异常，血氨及肌酐明显升高伴尿量减少，考虑肝衰竭合并肝性脑病及急性肾损伤。立即予输注血制品、降氨、改善肾血流、血液透析（CVVHDF模式）、血浆置换、抗感染等对症支持治疗。经积极处理，复查PT 20～24 s，APTT 50～60 s，凝血酶原活动度（PTA）30％～40％，凝血酶原时间国际标准化比值（INR）2.5～2.8，Fib 1.2～1.9 g/L，PLT（25～50）×10^9^/L，凝血酶-抗凝血酶复合物（TAT）、纤溶酶-α2纤溶酶抑制物复合物（PIC）均明显升高（TAT 11.7 µg/L，PIC 1.75 mg/L），TAT/PIC>5，提示高凝状态；血栓弹力图（TEG）总体呈现低血小板、低纤维蛋白原状态。术后第4天复查CT示门静脉右前支分支血栓形成并肝缺血，经多学科会诊评估血栓有进展风险，遂加用低分子肝素0.2 ml每日1次皮下注射（根据PT、APTT、TAT/PIC、TEG的变化调整剂量）。数日后，TAT及TAT/PIC水平明显下降，复查CT未见肝缺血灶进展。虽然患者凝血紊乱得到一定程度纠正，但意识及胆红素水平仍进行性恶化，遂于6月12日在全麻下行同种异体原位肝移植术［供肝来自于经严格脑死亡判定程序确认脑死亡的器官捐献者并由中国人体器官分配与共享计算机系统（COTRS）进行分配）］，术中出血1200 ml，共输注红细胞悬液10 U、新鲜冰冻血浆1000 ml、机采血小板1治疗量、凝血酶原复合物1200 U、纤维蛋白原2 g。术后2 h右膈下、肝门部引流管引流鲜红色血性液体约800 ml，HGB进行性下降，血乳酸8.9 mmol/l且呈上升趋势，出凝血检查示PT 21 s、APTT 65 s、Fib 1.22 g/L，考虑存在创面渗血，予目标导向血制品输注、抗纤溶、纠酸、复温等对症处理。术后5 h患者神志转清，血乳酸逐渐下降至正常水平，出凝血指标明显改善，TEG示凝血功能基本正常。术后每日行床旁超声见移植肝肝动脉、门静脉血运良好，6月15日患者顺利脱机拔管，同时尿量恢复，逐渐减停连续性肾脏替代治疗（CRRT）并转回普通病房。多次随访移植肝血运良好，肝肾功能均恢复正常，最终顺利出院。

**图1:**
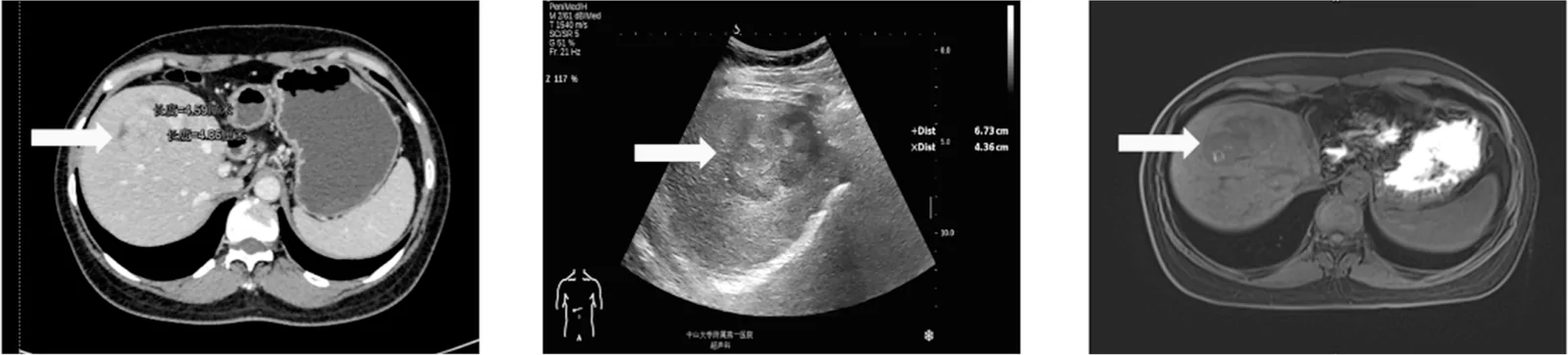
1例原发性肝癌患者肝切除术前影像学检查结果 **A** 增强CT；**B** 超声造影；**C** 普美显增强磁共振成像

## 讨论及文献复习

终末期肝病患者，病情重、进展快、病死率高，肝移植是其最为有效的治疗手段，随着技术及经验趋于成熟完备，肝移植围手术期各种并发症的发生率及死亡率均明显降低，但出血及血栓形成仍是围手术期较为常见且危害较大的并发症，据统计，肝移植患者围手术期凝血功能障碍可增加围手术期出血、血管并发症及再次手术的风险。因此应重视此类患者围手术期的凝血管理，以期减少并发症及提高术后生存率。

肝脏在凝血过程中起着至关重要的作用，肝功能衰竭时其合成的促凝和抗凝因子都减少，同时其代谢激活状态的凝血因子的能力也下降，使得凝血-抗凝-纤溶三者间形成的动态平衡被打破，取而代之的是这三者间另一个低水平的脆弱的平衡状态。这种脆弱的平衡极易被破坏，因此机体所表现出来的状态是多样且易变的，既可表现为高出血倾向，也可表现为高凝状态，甚至两者并存[6]。

本例患者肝移植术前出凝血指标多项异常，凝血处于极度紊乱状态：血小板减少，PT、APTT、INR均明显延长，Fib水平降低；TEG示血小板功能下降及纤维蛋白活性降低，符合肝衰竭患者的凝血特征；TAT明显升高且TAT/PIC>5，提示有血栓进展的高风险。增强CT证实患者门脉血栓形成合并肝缺血。经过TEG动态监测下合理调整抗凝方案使得门脉血栓未进行性进展；该患者术中术后创面渗血均较多，而新肝发挥作用需要一定时间，根据2023年欧洲创伤后大出血和凝血病管理指南，建议依据出凝血结果目标导向输注血制品及早期抗纤溶，同时因移植术后凝血和抗凝系统恢复速度的不同步极易导致术后高凝反应，需高度警惕矫枉过正导致移植肝血管并发症的发生，该患者术后根据出凝血结果的动态变化不断调整优化输注血制品的种类、剂量和比例，同时尽早启动抗纤溶治疗，同时辅以积极纠酸、复温，患者术后渗血情况得以迅速受控，且经术后多次复查移植肝超声未见肝血管血流异常。

由上述病例可见，加强对肝移植围手术期凝血功能的动态监测对我们的治疗策略有很强的指导价值。传统的实验室出凝血功能监测包括：①血管内皮系统检测，包括出血时间（BT）、血浆内皮素-1（ET-1）等；②血小板相关检测，包括血小板计数、血小板黏附试验、血小板聚集试验；③凝血系统检测，包括PT、APTT、INR、Fib等[7]；④抗凝血系统检测，包括蛋白C/蛋白S活性检测、抗凝血酶Ⅲ（AT-III）活性检测等；⑤纤溶系统检测，包括D-二聚体、纤维蛋白（原）降解产物等。虽然传统检测指标基本覆盖了整个凝血过程中的各个阶段，但单一指标只能反映凝血过程中某一阶段或某种凝血产物，不能阐明凝血的全过程，因此有一定的局限性，并不能准确判定出血或形成血栓的风险。近年来，TAT、PIC等在凝血和纤溶系统反应起始阶段产生的血栓早期分子标志物已用于疾病的早期诊断[8]。

把握肝移植围手术期各时期凝血功能的变化规律，结合上述监测指标，可以更好地指导临床实践，减少围手术期并发症的发生并改善预后[9]。

术前肝移植受者因肝功能衰竭、继发脾功能亢进及血小板生成素（TPO）生成减少，最终导致血小板数量减少和功能异常，除此之外，肝衰时大部分凝血因子（除外VWF）、抗凝因子（AT-Ⅲ、蛋白C及蛋白S等）生成减少、同时α-2纤溶酶抑制物（α-2 AP）、凝血酶激活的纤溶抑制物（TAFI）、FXIII水平下降及对组织型纤溶酶原激活剂（t-PA）等的清除能力减弱共同导致不同程度的纤溶亢进以及门脉高压[10]，致使术前出血风险高；此外，此类患者同时存在严重的内皮功能障碍，包括FⅧ和VWF在内的诸多凝血因子水平明显升高，极易导致高凝状态，而灭活VWF必需的ADAMTS13的生成减少、凝血酶生成增加等因素进一步加剧了高凝风险。因此术前凝血功能可能表现为低水平的脆弱的平衡、失衡的出血或高凝状态，呈现出高度的不可预测性，鉴于常规手段既无法体现蛋白C和蛋白S的活性，又无法评估凝血因子、血小板和其与血管内皮之间的相互作用，通过常规的出凝血监测手段难以进行准确的描述与评估。因此，能提供整个凝血过程动态信息的凝血功能检测手段——黏弹性测试在围手术期管理中具有重要作用[11]，常用的黏弹性测试包括TEG和旋转血栓弹性测量（ROTEM）。目前，已有部分指南和共识建议采用TEG评估肝病患者的出凝血状态，并用于指导肝移植围手术期血液制品的输注[10],[12]–[13]。针对术前严重的凝血功能紊乱，可结合TEG并视具体情况补充诸如新鲜冰冻血浆（FFP）、纤维蛋白原、凝血酶原复合物等，但这些仅能短时间改善患者的凝血状态，不能从根本上逆转凝血紊乱，因此临床实践中应密切关注患者有无出血倾向，适时、恰当的补充相应凝血因子。

因手术所处时段不同，影响凝血系统的因素亦有差别。在无肝前期，该阶段主要包括全身麻醉和全肝切除。一方面因原发病导致的低凝状态仍然存在，另一方面麻醉液体复苏所致稀释性凝血障碍及术中出血所致消耗性凝血障碍都极易使脆弱的凝血功能迅速恶化[14]，导致难以控制的术中出血甚至DIC的发生。因此无肝前期以凝血因子缺乏为主要表现。该期适量使用血管活性药物可有效减少复苏液体量，同时妥善的术中止血、根据凝血功能指标的动态变化针对性输注凝血药物以尽量控制静脉容积压力减少出血风险都对该阶段凝血功能的稳定大有裨益。在无肝期，患者体内本就脆弱的凝血平衡遭到广泛打击，该阶段患者全肝被完全移除，且门脉与下腔静脉血流被完全阻断。因此在该阶段，肝功能的完全丧失致使凝血因子水平迅速下降及无法清除t-PA而导致纤溶增强，同时因回心血流阻断，脾功能亢进进一步破坏血小板及因肠道淤血致使肠道菌群易位和内毒素入血加重凝血障碍[15]。因此，无肝期以凝血因子显著减少、血小板下降、原发性纤溶亢进为主要表现。此期应尽量减少无肝期的时长，以尽快恢复凝血因子的合成，减少纤维蛋白溶解和内毒素增加为主要目标。值得注意的是，若在该时期输注过多的促凝物质有引发微血管血栓形成甚至肺梗死的风险，应继续动态监测凝血功能的变化并根据术中出血情况适时输注凝血药物。在新肝期，该阶段低温新肝发生肝窦内凝血会消耗大量凝血因子，随着门脉和下腔静脉的开放，体内会有一过性的高肝素血症，同时因新肝释放的大量t-PA无法被及时清除导致纤溶亢进[16]。新肝功能在复流后逐渐恢复并开始启动凝血因子合成。可以动态监测TEG和凝血功能，酌情使用凝血药物以纠正凝血紊乱。除上所述机制外，术中低体温、低钙、高乳酸等因素均会加重出血风险，因此加强术中体温监测和主动保温、及时纠酸和低钙血症可有效改善凝血功能[17]–[18]。

术后早期，因移植肝功能尚在逐步恢复的过程中，一般而言，术后第1天凝血因子合成功能即可恢复，而抗凝因子合成功能的恢复较慢，一般术后2周左右才得以恢复[19]；加之术后早期合并有因术中出血导致凝血因子消耗过多、脾亢未改善致血小板减少及术中残留肝素等多种因素，整体呈现低凝状态。但随着血小板水平的回升、免疫抑制剂的使用等多种因素的共同作用，后续可能逐渐转变成高凝状态，需密切关注移植肝血流情况，及早识别高凝风险并预防性抗凝可减少移植肝血管内血栓形成概率，进而有效减少移植物失功和二次移植的发生率[20]。

随着肝移植手术的普及，围手术期凝血管理的重要性愈发突出。由于不同病程阶段病理生理机制的差异，围手术期凝血功能变化复杂且难以预测，但通过动态监测可实现有效干预。肝移植患者常面临术前血小板与凝血因子缺乏、术中低凝与纤溶亢进以及术后凝血功能恢复不全等问题，因此科学管理尤为关键。

本例患者在肝部分切除术后出现严重肝功能不全、急性肾损伤及凝血功能异常，同时在凝血时间延长的基础上出现了门静脉血栓。虽然患者在TEG与血栓四项等动态监测下抗凝治疗，门静脉血栓有所改善，但肝功能仍进行性恶化，最终行肝移植术。术中及术后早期患者出血较多，但通过目标导向输注血制品及抗纤溶治疗，凝血功能恢复，术后肝肾功能逐渐恢复正常，最终顺利出院。
